# Prevalence and healthcare burden of inappropriate antimicrobial treatment in patients at high risk of complications from acute respiratory infections: a scoping review

**DOI:** 10.3389/fmed.2025.1533797

**Published:** 2025-05-16

**Authors:** Cate Polacek, Tristan T. Timbrook, Chendi Cui, Zoe Heins, Ning A. Rosenthal

**Affiliations:** ^1^Premier Inc., Charlotte, NC, United States; ^2^Global Medical Affairs, bioMérieux, Inc., Salt Lake City, UT, United States; ^3^College of Pharmacy, University of Utah, Salt Lake City, UT, United States

**Keywords:** acute respiratory infection, disease burden, high risk patients, risk factors, burden of illness, antimicrobial prescribing

## Abstract

**Background:**

Evidence of inappropriate antimicrobial treatment for acute respiratory infections (ARIs) is well-established in literature. However, comprehensive evaluations of inappropriate antimicrobial use and associated clinical and economic outcomes for patients at high risk of complications from ARIs are lacking. This scoping review described the prevalence of inappropriate antimicrobial use and its healthcare burden in this patient population.

**Materials and methods:**

We queried Medline and CINAHL databases using keywords related to antimicrobials for ARIs in high-risk patients, and included United States studies reporting prescribing patterns, outcomes, adverse events, and costs.

**Results:**

Our search yielded 3,383 studies after de-duplication, from which 482 were selected for full-text evaluation based on exclusion criteria, resulting in 32 papers analyzed that included relevant information on high-risk populations. The analysis suggested that patients at high risk for complications experience improper prescribing of antimicrobials for ARIs, which is associated with higher direct and indirect costs, increased health care resource utilization, higher incidence of adverse events, and more severe disease complications.

**Conclusion:**

Areas for improving care for this patient population include identifying patients at risk of severe disease and complications from ARIs and following evidence-based protocols for testing, treatment and follow-up to minimize the risk of developing adverse events, antibiotic-resistance, and severe complications.

## 1 Introduction

Acute respiratory infections (ARIs) are common in both children and adults and are among the top three diagnoses in outpatient settings in the United States (US) (1). Although most ARIs are minor, they may lead to severe, sometimes life-threatening complications, especially in high-risk patients including infants, the elderly, and individuals with comorbid or immunocompromising conditions. A systematic review highlighted that globally, ARIs accounted for more than half of deaths in adults aged 60 years or older in 2015 (2). The COVID-19 pandemic further underscored the increased risk of developing more severe clinical outcomes as a result of COVID-19 infection among people who were older or had pre-existing conditions such as diabetes, chronic lung disease, cardiovascular disease, kidney disease, obesity, and immunocompromising conditions (3, 4). Similarly, those with asthma or congestive heart failure may develop more severe infection from respiratory syncytial virus (RSV) including bronchiolitis and pneumonia (5). Influenza may also potentially exacerbate pre-existing conditions, especially asthma and chronic obstructive pulmonary disease (1).

Acute respiratory infections are associated with substantial economic burden due to cost drivers such as hospitalization and emergency department visits. In 2015, the annual total economic burden of influenza was estimated at $11.2 billion in the United States, with direct medical costs of $3.2 billion and indirect costs of $8.0 billion (6). Since the first case of COVID-19 was confirmed in the United States in January of 2020, COVID-19 has caused more than 1.2 million deaths as of September of 2024 nationwide (7), and the direct and indirect economic loss due to COVID-19 pandemic is tremendous (8).

These burdens are also seen in high-risk populations. Among United States adults hospitalized with influenza between 2016 and 2023, those who needed mechanical ventilation or intensive care unit admission or both had higher average hospitalization costs if they were 50–64 years old or had complications related to influenza infection (9). Another study noted that older age and comorbidities were associated with nine times higher odds and 2–3 times higher odds of hospitalization for influenza, respectively (10). The Centers for Disease Control and Prevention (CDC) also reported that every year, RSV results in approximately 2.1 million outpatient visits and 58,000–80,000 hospitalizations among children younger than 5 years old, as well as 100,000–160,000 hospitalizations among adults 60 years and older (11). RSV treatment costs for infants is estimated at $709.6 million annually (12), while RSV-associated costs for adults is estimated at $42,179 for hospitalization, $4,409 for emergency department care, and $922 for outpatient care during the acute phase of illness (13).

Increased understanding of the short-term and long-term effects of ARIs in high-risk populations may guide future research and interventions for managing respiratory infections in this population. While systematic reviews on inappropriate antibiotic prescribing have been published in the literature (14–18), an up-to-date summary of the prevalence of inappropriate antimicrobial use and its associated clinical outcomes and economic burden for high-risk populations is lacking. This scoping review aimed to summarize up-to-date evidence and knowledge gaps in inappropriate antimicrobial use, including antibiotics and antivirals, in treating ARIs and their related clinical and economic impacts on high-risk populations.

## 2 Materials and methods

We conducted a scoping literature review and reported results according to the Preferred Reporting Items for Systematic Reviews and Meta-Analyses Extension for Scoping Reviews (Supplementary Table 1) (19, 20). The Medline and CINAHL databases were searched to identify peer-reviewed studies published between January 2014 and September 2024 reporting on inappropriate antibiotic and antiviral use and their respective clinical and economic consequences in patients with ARIs who were at high risk of complications. Specific ARIs used in the search included infections due to influenza, adenovirus, coronavirus, metapneumovirus, rhinovirus, enterovirus, and RSV.

### 2.1 Literature search strategy

Studies were searched using terms related to respiratory tract infections (RTI) and etiologies, antibiotics, antivirals, inappropriate use, healthcare expenditures and outcomes in high-risk populations (Supplementary Table 2). Inclusion criteria for studies included those published in the last 10 years, peer-reviewed publications for original investigation, study population within the United States, and written in English. Duplicate articles and certain article types (case studies, case reports, comparative drug clinical trials, studies with sample size fewer than 100 patients) were excluded. Titles and abstracts of the remaining articles were reviewed, and full-text articles were obtained for further screening.

### 2.2 Data extraction

Information extracted from articles included, when available: study type, time period or dates of study; data sources, population size, age range, genders, and race/ethnicity; follow-up period; outcome measurements; and key findings and statistics. One reviewer (C.P.) performed the initial search, abstract and full text screening, and data extraction. Following data extraction, two reviewers (C.P., C.C.) discussed analysis of queries on findings and reviewed additional articles as needed. All data extraction was recorded using Microsoft Excel 365 (Microsoft Corporation).

## 3 Results

### 3.1 Included studies

**FIGURE 1:**
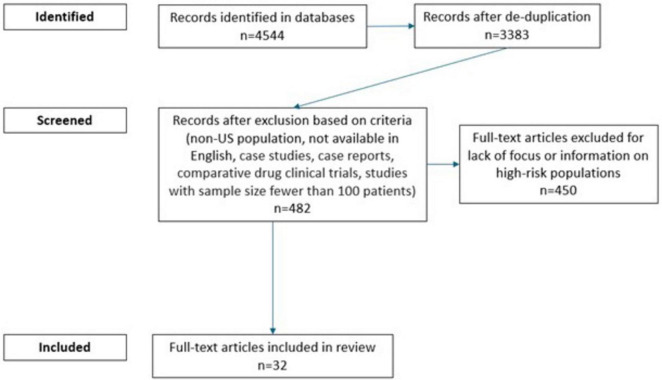
Flow diagram for article selection.

The literature search resulted in 3,383 unique studies after de-duplication. After applying exclusion criteria, 482 were included for full-text evaluation. Following full-text screening, 32 papers meeting selection criteria were included in the review (Figure 1). Of these, nine articles reported on antibiotics, while 23 articles reported on antivirals in high-risk populations. Additionally, four articles reported on ARIs in general, 19 articles reported on influenza, four articles reported on pneumonia, and five articles reported on COVID-19 in high-risk populations. A summary of findings can be found in Tables 1, 2.

**TABLE 1:** Summary of findings - Inappropriate antibiotic use in treating acute respiratory infections (ARIs) in high-risk patients.

References	Extracted key findings
**Pneumonia**	
Singh et al. (21)	• 55.4% of all ARTI outpatients were treated with antimicrobials • A lower comorbidity score was significantly associated with receiving antimicrobials (*p* < 0.0022)
You et al. (22)	• Patients receiving vancomycin were younger, more likely to have renal disease and sepsis, more likely to be admitted to the ICU, and more likely to have received antibiotics within the last 90 days than those who did not receive vancomycin.
Leyenaar et al. (23)	• Children with complex chronic conditions were: • Two to five times more likely to receive initial antibiotic coverage against MRSA, anaerobic organisms, and *Pseudomonas* spp. • More likely to have antibiotic coverage expanded on or after second day of hospitalization. • More likely to have antibiotic coverage broadened.
Amirault et al. (27)	• Compared with older children, infants < 90 days had more laboratory testing, and wide variation in testing across hospitals. • Antibiotics were administered less often for younger infants compared to older children (74.1% versus 84.4%; *P* < 0.001).
Wang et al. (29)	• 161 patients in the pre-CDS group and 119 patients in the post-CDS group included in the study. • HIgh-risk criteria for consideration in post-CDS group included nursing home/long-term care facility residence, chemotherapy, chronic hemodialysis, immunosuppressive disease or therapy • Significant improvement in the selection of appropriate antibiotics in the post-CDS group (31.9% versus 65.3%, *P* < 0.0001) with no significant differences in duration of antibiotics, intubation rates, vasopressor initiation, length of stay, mortality or 30 days readmission.
**Influenza**	
Hartnett et al. (24)	• United States cohort - 280 influenza-positive and 120 RSV-positive patients • Core risk factors (CRFs) for severe disease - age ≥ 65 years, chronic heart or renal disease, chronic obstructive pulmonary disease, or asthma) • RSV patients were older (mean: 63.1 vs. 59.7 years) and a higher proportion had CRFs (87.5% vs. 81.4%) • At 3 months post-discharge, more RSV patients with CRFs reported use of antibiotics, antitussives, bronchodilators, and inhaled and systemic steroids versus those with influenza and CRFs.
**Respiratory tract infections - general**	
Barlam et al. (25)	• Current tobacco users were overprescribed antibiotics more often than non-smokers.
Jones et al. (26)	• 51.9% had a cardiovascular comorbid condition, 24.1% had a pulmonary comorbid condition • The number of patient comorbid conditions had no association with antibiotic prescribing for respiratory infection • There was a substantial increase in macrolide prescribing, which has potential cardiotoxicity, despite the number of Veterans who had cardiovascular comorbid conditions
Cerone et al. (28)	• Infants with clinical suspicion for RVI evaluated for late-onset sepsis • 29 (8%) had a respiratory virus detected • RSV (14 of 29; 48%) was the predominant virus detected. • Antimicrobial therapy was withheld or discontinued on most infants with a virus detected (18 of 29; 62%) and in the majority where there was no confirmed bacterial co-infection (18 of 20; 90%)

**TABLE 2:** Summary of findings - Underuse of antiviral treatment for patients at high risk of complications .

References	Extracted key findings
**Influenza**	
Shi et al. (33)	• Risk of complications was higher in children with pre-existing conditions than otherwise healthy children. • Antiviral treatment reduced the 30 days risk of complications, hospitalization, ED visits, and outpatient visits versus no treatment. • 30 days risks were further reduced by early treatment (within 2 days of diagnosis). • In children aged > 1 year, 7.38% received antiviral treatment during pre-pandemic seasons and 33% during the pandemic season. • Healthcare resource utilization was only reduced by early treatment.
Fowlkes et al. (36)	• In age groups recommended to receive empiric antiviral treatment for suspected influenza, 11% of children < 2 years and 23% of adults ≥ 65 years received a prescription.
Havers et al. (37)	• Among high-risk patients, 7% received an antiviral prescription, including 15% of high-risk patients who presented early. • Among high-risk patients with ARI, 14% were prescribed an antiviral compared with 20% of patients with influenza-like illness (ILI). • Among high-risk patients who presented early, the proportion treated with antiviral medications was highest among pregnant women and those who were morbidly obese. • 6% of children aged < 2 years who presented early received an antiviral prescription. • No children aged < 2 years with chronic medical conditions were prescribed an antiviral medication, including 30% who presented early. • 24% of high-risk patients with lab-confirmed influenza were prescribed an antiviral medication - the proportion was higher among those who presented early and was higher than high-risk patients without lab-confirmed influenza who presented early.
Havers et al. (38)	• Among ARI patients at high risk for influenza complications presenting to care ≤ 2 days from symptom onset, 19% were prescribed an antiviral medication.
Lindegren et al. (39)	• Of hospitalized patients prescribed antivirals, 87% had underlying high-risk conditions. • Approximately 29% of patients were hospitalized within 2 days of symptom onset, yet antiviral use was low. • Antiviral treatment was more common in those age 50–64 years compared to 65 years and older.
Appiah et al. (40)	• Among patients with high-risk conditions, 86% received antivirals compared with 77% without. Across all seasons, treatment increased over time from 76% to 90% in patients with high-risk conditions. • Among children aged < 18 years with high-risk conditions, 77% were treated compared with 87% adults with high-risk conditions. • Among treated patients, 83% had a high-risk condition compared with 72% of those not treated.
Stewart et al. (42)	• Among high-risk outpatients with ARI who presented to care within 2 days of symptom onset, 15% were prescribed an antiviral medication, including 37% of those with rRT-PCR–confirmed influenza. • 40% of high-risk outpatients with influenza presented to care early. • Earlier presentation and fever was associated with antiviral treatment, although 25% of high-risk outpatients with influenza were afebrile. • Empiric treatment of 4 high-risk outpatients with ARI was needed to treat 1 patient with influenza. • 40% of high-risk outpatients with lab-confirmed influenza presented to care early; 37% of these received a prescription for an antiviral medication. • Older adults with influenza were least likely to present within 2 days compared to all other age groups and most likely to present > 4 days after symptom onset.
Mossad (43)	• 15% of high-risk patients were prescribed anti-influenza medications within 2 days of symptom onset, including 37% in those with lab-confirmed influenza. • Fever was associated with an increased rate of antiviral treatment, but 25% of high-risk outpatients were afebrile. • Empiric treatment of four high-risk outpatients with ARI was needed to treat one patient with influenza
Williams et al. (44)	• Outpatient providers reported prescribing antivirals to those with flu-like symptoms for 31% of children < 2 years, 23% of children 2–5 years, 37% of pregnant patients, and 74% of other patients at high risk.
Biggerstaff et al. (46)	• Patients aged 18–64 years with asthma or heart disease, disability, and financial barriers to healthcare access were more likely to report ILI. Similar associations were seen in those older than 65 years. • 40% with ILI sought care, and 14% who sought care received influenza antiviral treatment. • Treatment was not more frequent in patients with high-risk conditions, except those aged 18–64 years with heart disease. • Of patients at high risk for influenza complications, self-reported ILI was greater but receipt of antiviral treatment was not
Best et al. (54)	• Patients ≥ 66 years old with an influenza diagnosis in outpatient setting. • Treated patients received antivirals 2 days from index. • Untreated patients had no antivirals 6 months post-index. • All-cause mortality within 6 months from index diagnosis was 1.6% among treated versus 4.3% among untreated patients. • Rates of all-cause inpatient hospitalizations during follow-up were 13.9% versus 22.7%. • Respiratory-related hospitalizations were 4.2% versus 9.0%. • Mean (SD) total all-cause and respiratory-related costs were $9,830 ($18,616.0) and $900 ($4016.4) among treated, respectively, versus $13,207 ($24,405.1) and $2,024 ($7,623.7) among untreated, respectively. • All differences were statistically significant (*p* < 0.001).
Biggerstaff et a. (51)	• Receipt of influenza antiviral medication was higher among those with diabetes but lower among those who were disabled. • Patients who sought care within 2 days were more likely to receive influenza antiviral medication than those who sought care later.
Fuller et al. (52)	• Patients treated > 2 days after admission had more comorbidities than patients treated within 2 days of admission. • Patients never treated during hospitalization had more documentation of CV and other diseases than treated patients. • More severe endpoints were observed in patients never treated or patients treated > 2 days after admission than in patients treated earlier.
Hamdan et al. (47)	• Clinicians provided antiviral treatment for 52% of patients with a positive influenza test. • Factors associated with testing included neuromuscular disease, immunocompromised status, age, private only versus public only insurance, and chronic lung disease. • Factors associated with antiviral treatment included neuromuscular disease, immunocompromised state, duration of illness, and chronic lung disease.
Suryaprasad et al. (50)	• RIDT results had a stronger association with receipt of early antivirals than did the presence of risk factors for H1N1pdm09 complications.
Adams et al. (35)	• Underlying medical conditions included chronic lung disorder, chronic metabolic disorder, blood disorder, cardiovascular disorder, neurologic disorder, immunocompromised condition, renal disease, gastrointestinal or liver disease, rheumatologic, autoimmune, or inflammatory conditions, hypertension, obesity, pregnant. • Received influenza antiviral treatment (*p*-value 0.44). • Patients with influenza and SARS-CoV-2 coinfection 17 (53.1%). • Patients with only influenza 326 (60.0%).
Antoon et al. (34)	• Dispensing rates ranged from 4.4 to 48.6 per 1,000 enrolled children. • Treatment rates highest among children 12–17 years of age, during the 2017 to 2018 influenza season, and in the East South Central region. • Guideline-concordant antiviral use among young children (< 2 years of age) at a high risk of influenza complications was low (< 40%). • Inflation-adjusted cost for prescriptions was $208,458,979. • Median cost ranged from $111 to $151.
Corral et al. (55)	• Across influenza seasons, patients with CVD and influenza who received antiviral treatment had fewer all-cause ED visits, respiratory-related HRU, respiratory-related outpatient and ED visits, CVD-related HRU, heart failure-related HRU visits, and kidney failure-related HRU 180 days post-treatment fill date than CVD patients untreated for influenza. • CVD patients treated with antivirals also had fewer all-cause inpatient, outpatient, and ED visits and days of stay and fewer mean respiratory-related outpatient and ED visits. • HRU patterns were consistent over time and across influenza seasons. • Treated CVD patients had lower all-cause outpatient costs post-treatment fill date than CVD patients untreated for influenza.
**COVID-19**	
Bajema et al. (48)	• Among Veterans with documented COVID-19–related symptoms in the 30 days preceding a positive SARS-CoV-2 test, 5.5% received any COVID-19 pharmacotherapy. • Untreated Veterans had a median age of 60 years and a median of three underlying medical conditions. • Veterans receiving any treatment were more likely to be older and have a higher number of underlying conditions.
McGarry et al. (41)	• Residents treated for COVID-19 among nursing homes had an overall oral antiviral or monoclonal antibody treatment rate of 17.8%. • Only one in four nursing home residents with COVID-19 had been treated with evidence-based antiviral treatments by the end of 2022. • More than 40% of nursing homes reported never administering any oral antiviral or monoclonal antibody treatment.
Monach et al. (49)	• Of Veterans with mild to moderate infection at high risk for progression due to underlying conditions who did not receive an antiviral drug, 20% were offered treatment but declined; 80% were not offered treatment. • Among patients not offered treatment, provider reasons included symptom duration of > 5 days, concern about possible drug interactions, or absence of symptoms. • Among nearly one half of these patients, no reason other than mild symptoms was given. • Among 55.8% of those patients, follow-up consisted of telephone calls to provide test results and ask about symptoms, with no documentation of offering treatment.
Mehta et al. (45)	• 8,754 (6.3%) received hydroxychloroquine, 29,272 (21.2%) received remdesivir, 53,909 (39.1%) received dexamethasone. • 19.8% of patients had a score of one, 11.9% a score of two, 8.4% a score of three, 25.8% a score of four or greater comorbid conditions assessed by the Charlson Comorbidity Index. • Dexamethasone was more commonly used among older adults (45.3% among those aged ≥ 75 years vs. 20.2% among those aged 18 to 34 years), males (41.3% vs. 36.8%), non-Hispanic White compared with non-Hispanic Black persons (45.4% vs. 33.6%), and those requiring mechanical ventilation (55.9%vs. 37.3%). • Similar patterns were seen with remdesivir use, with greater use among patients who were older, male, non-Hispanic White, or obese and those with more severe COVID-19 or comorbid illness. • Among mechanically ventilated patients, dexamethasone or other glucocorticoids was more frequent among those with diabetes than those without (56.2% vs. 42.6%).
Gentry et al. (56)	• Any hospitalization or death within 30 days of diagnosis was significantly lower in patients receiving oral antiviral therapy compared to those who did not. • Difference driven largely by fewer deaths in oral antiviral group. • No significant difference in rate of intensive care requirement.

### 3.2 Inappropriate antibiotic use in treating ARIs among high-risk patients

Our review found discrepancies between clinical practice patterns and guideline recommendations, and that reasons for overuse may include potential demographic influences, provider habits, and lack of protocols for some populations.

Overuse of antibiotics was found in a variety of patient populations (21). For example, a retrospective observational study (22) of 768 patients hospitalized with pneumonia found that while only 2.6% had an MRSA-positive culture, 38.3% were given vancomycin, despite current guidelines discouraging methicillin-resistant *Staphylococcus aureus* (MRSA) coverage. Patients treated with vancomycin were significantly younger, more likely to have renal disease and sepsis, had a higher probability of ICU admission, and were more frequently prescribed other antibiotics in the preceding 90 days compared to those who did not receive vancomycin. A study of children with pneumonia observed that those with chronic health conditions were more likely to receive antibiotic escalation during their hospital stay (23). Another study (24) of patients with RSV or influenza noted that of those with risk factors for severe disease, such as being older than 65 years, or having chronic heart or renal disease, chronic obstructive pulmonary disease, or asthma, RSV infection was associated with disease burden and medical resource usage, including antibiotics, equivalent to or greater than that of influenza, even 3 months post-discharge from hospital. Finally, a retrospective analysis of RTI visits using data from the National Ambulatory Medical Care Survey and the National Hospital Ambulatory Medical Care Survey noted that tobacco users were overprescribed antibiotics for ARIs compared to non-smokers (25).

Interestingly, provider habits may play a role and might override other risk factors. One study of Veterans pointed this out (26), noting that providers had a tendency to choose the same treatment regardless of patient or clinic characteristics. Although 51.9% had a cardiovascular comorbid condition and 24.1% had a pulmonary comorbid condition, these risk factors did not appear to have an effect on antibiotic prescribing for ARIs. Indeed, there was a substantial increase in macrolide prescribing, even for Veterans with cardiovascular comorbid conditions, despite the potential cardiotoxicity of therapy.

Diagnostic uncertainty, coupled with a lack of guidelines for testing and treatment, may also result in potential “cautious overprescribing.” One study of community-acquired pneumonia (CAP) in children < 90 days old to 5 years old found differences in diagnostic testing rates, antibiotic administration, and outcomes and noted that in addition to lack of guidelines, clinicians may have difficulty differentiating CAP from other lower respiratory tract infections (LRTI) in young infants (27). Conversely, there were several examples in the literature demonstrating that testing and other tools, such as order sets, may help avoid or correct overprescribing unnecessary antibiotics in high-risk populations. A study of infants with potential respiratory viral infection (RVI) who were evaluated for late-onset sepsis found that 8% actually had a virus detected (RSV predominantly) (28). Unnecessary antimicrobial therapy was then withheld or discontinued in most patients with no confirmed bacterial co-infection (90%) and in 62% of infants with a virus detected. Another study implemented a clinical decision support (CDS) alert that included high-risk criteria, such as nursing home or long-term care facility residence, chemotherapy, chronic hemodialysis, immunosuppressive disease or therapy, when selecting medications (29). Significant improvement of appropriate antibiotic prescribing was seen in the post-CDS group.

### 3.3 Underuse of antivirals in treating ARIs among high-risk patients

The CDC (30, 31) and the Infectious Disease Society of America (IDSA) (32) recommend testing and early antiviral treatment for people who have flu, suspected flu, or COVID and have a higher risk of serious complications. However, we noted several studies demonstrating that antiviral treatment was underprescribed, particularly among young children (33–35), older adults (36–41), and among those in high-risk groups (37, 42–45). A community survey administered via the CDC Behavioral Risk Factor Surveillance System (46) showed that, except for individuals aged 18–64 with heart disease, patients with high-risk conditions did not receive increased treatment for influenza-like illness (ILI). Despite a higher self-reported incidence of ILI among patients at high risk for influenza complications, antiviral treatment rates did not correspondingly increase. Another study (47) found that clinicians provided antiviral treatment for 52% of patients with a positive influenza test, and factors associated with prescribing antiviral treatment included neuromuscular disease, immunocompromised state, duration of illness, and chronic lung disease. A cohort study of (48) United States Veterans who had risk factors for severe COVID-19 also noted that pharmacotherapy was underused. Among those with documented COVID-19–related symptoms in the 30 days preceding a positive SARS-CoV-2 test, only 5.5% received any therapy. Those who were untreated had a median age of 60 years and a median of three underlying medical conditions. A separate study of (49) Veterans with mild to moderate COVID-19 infection at high risk for progression due to underlying conditions such as organ transplantation or hematologic malignancies who did not receive an antiviral drug found that among patients not offered treatment, provider reasons included symptom duration of > 5 days (22.7%), concern about possible drug interactions (5.7%), or absence of symptoms (22.7%). For about one half (48.9%) of these patients, no reason was given aside from mild symptoms.

Testing may also be a factor in prescription of antivirals. One study (50) noted that rapid influenza diagnostic testing results had a stronger association with receipt of early antivirals than the presence of risk factors for H1N1pdm09 complications (age 5 years or high-risk medical conditions). Another study found that factors associated with testing included neuromuscular disease, immunocompromised status, age, private only versus public only insurance, and chronic lung disease (47).

The timing of presentation may also influence treatment. One study demonstrated that only 7% of high-risk patients received an antiviral prescription, including 15% who sought care early (37). Among high-risk patients presenting early, the highest proportion treated with antiviral medications was pregnant women (43%) and those with morbid obesity (25%). Conversely, only 6% of children aged < 2 years presenting early received an antiviral prescription, and no children aged < 2 years with chronic medical conditions were prescribed an antiviral medication, including 30% who presented early. Another study (51) showing that receiving antiviral medication for influenza was higher among those with diabetes (46%) but lower among those who were disabled (18%), also noted that patients who sought care within 2 days (55%) were more likely to receive influenza antiviral medication than those who sought care later (35%). Another study (52) noted that patients treated more than 2 days after admission had more comorbidities than patients treated within 2 days of admission, and that patients who were never treated during hospitalization had more documented CV and other diseases than treated patients.

### 3.4 Clinical and economic outcomes for patients in high-risk populations

Several studies demonstrated that clinical and economic outcomes are worse for patients at high risk of complications, especially if they are untreated, not treated early, or if treatment is delayed (9, 53, 54). These trends were seen for several ARIs and for both adults and children. One retrospective cohort study (33) of antiviral treatment in children revealed that during the 2006–2010 influenza seasons, the risk of complications was significantly higher in children who had preexisting conditions, especially respiratory conditions such as asthma or cystic fibrosis. In children older than a year, 7.38% received antiviral treatment during pre-pandemic seasons, and 33% received treatment during the during the 2009–2010 pandemic season. Those who received antiviral treatment had a reduced risk of complications, hospitalization, ED visits, and outpatient visits versus no treatment within 30 days. Risks were further reduced with early treatment within 2 days of diagnosis. Healthcare resource utilization (HRU) was also reduced with early treatment. A retrospective claims analysis of (55) commercial and Medicare databases during three influenza seasons that compared outcomes and costs in patients with cardiovascular disease (CVD) found that who received antiviral treatment for influenza had fewer all-cause ED visits or respiratory-related outpatient and ED visits; and less respiratory-related, CVD-related, heart failure-related, and kidney failure-related HRU 180 days after treatment fill date than patients with CVD who were not treated. Patients treated with antivirals also had a lower mean number of all-cause inpatient, outpatient, and ED visits, and fewer days of stay or mean respiratory-related outpatient and ED visits, as well as lower all-cause outpatient costs 180 days after treatment fill date. Another study highlighting the importance of rapid treatment found that for older adults with flu, prompt antiviral treatment was associated with lower rates of mortality and acute complications, reduced hospitalization, and lower healthcare costs (54).

A retrospective descriptive study of (52) an HCA Healthcare electronic medical record dataset that was part of the FDA’s Sentinel System showed that hospitalized patients with chronic respiratory disease, CVD, liver or renal disorders, immune disorders, diabetes, obesity, hematological disorders, or who were smokers had more severe endpoints (death, mechanical ventilation, or ICU admission) if they were never treated with antivirals for influenza or were treated more than 2 days after admission. We also found a study that highlighted improved outcomes in patients when given appropriate antivirals – United States Veterans with immunocompromised conditions who had COVID and were given antivirals had lower incidences of hospitalization or death within 30 days of diagnosis (56).

## 4 Discussion

This study sought to evaluate inappropriate antibiotic and antiviral use and their respective clinical and economic consequences in patients with ARIs who have a high risk of complications. Results demonstrated that factors such as age, race, ethnicity, and comorbid conditions raise a patient’s risk of developing complications from ARIs. There is a high prevalence of inappropriate use and overuse of antibiotics and underuse of antivirals (57), even in instances in which therapy is contraindicated due to a co-morbid risk factor (26). These factors may lead to increased LOS in hospitals and higher costs of care associated with side effects, adverse events, and prolonged illness (58, 59).

Diagnostic uncertainty is a major contributor to inappropriate treatment (60). As viral and bacterial etiologies of infection overlap in signs and symptoms of presentation, overprescribing antibiotics, underprescribing antivirals, and related unnecessary healthcare expenditures often occur (60, 61). One tool for improving patient management in ARIs is rapid multiplex PCR diagnostics (62). A recent systematic review and meta-analysis reflected a 25% increase in appropriate neuraminidase inhibitor (NAI) use, 50% improvement in infection control, and approximately 1 day decrease in hospital LOS (est. United Statea $2 873 cost avoidance) with rapid multiplex PCR respiratory testing (62). The potential impact of rapid multiplex PCR testing on improving patient care management has been corroborated in other non-respiratory (i.e., meningitis and bloodstream infections) systematic reviews, particularly with a consistency of effect on decreasing length of stay by 1 day or more related to streamlining patient management and encounters (63, 64). The clinically actionable turnaround time and improved diagnostic yield translating into improved management has led guidelines to support the routine use of multiplex respiratory PCR panels (65, 66). While the impact of rapid multiplex panels in improving time to effective therapy and avoidance of unnecessary therapy is often noted in the literature, the utility for improving outcomes with NAI use has not been a significant focus. The utility of rapid multiple respiratory PCR toward clinical outcomes through driving early appropriate therapy for influenza patients is likely as research has reflected early administration (less than 6 h from admission) of NAIs for influenza is associated with decreased length of stay and mortality (67–69). Similarly, among high-risk and unvaccinated COVID-19 patients, therapy within the first 3 days of symptom onset has been associated with decreased hospitalizations and mortality (70).

Several studies demonstrate a trend of infrequent testing to verify type of infection, even though testing has demonstrated cost-effectiveness and decreased inappropriate antibiotic use (47, 50, 57). Yet guidelines recommend considering testing to aid diagnosis and treatment decisions and to ensure more careful use of antibiotics to avoid adverse events and exacerbation of treatment-resistant pathogens (61, 71–74). IDSA recommendations (61) note that factors such as illness severity, symptom duration, comorbidities, and net state of immunosuppression should be considered when determining whether to test for infection. Additionally, IDSA and Centers for Disease Control and Prevention (CDC) influenza testing algorithms for adults and children suggest testing and then treating is preferable to empiric treatment in cases of moderate disease prevalence or moderate to high severe disease risk. IDSA recommendations also suggest that multiplex viral nucleic acid amplification tests (NAAT) (potentially combined with bacterial NAAT) “makes clinical sense” for immunocompromised and critically ill patients with pneumonia and for patients with exacerbated airway disease (61). Providers and practice sites may benefit from additional education on these guidelines and revision of current policies to address clinical practice alignment with guidelines and improve appropriate antimicrobial prescribing.

Timely identification of causal pathogens of ARIs may help guide appropriate disease management strategies and may prevent hospitalizations and save lives, especially for patients with a higher risk of complications (75–77). The COVID-19 pandemic illustrated the urgent need for timely and accurate identification of infectious pathogens to help patients begin appropriate treatment early and to prevent potentially serious disease and complications (78, 79).

### 4.1 Limitations

This study has several limitations. First, the studies included were limited to high-risk populations in the United States and may not reflect global or general population trends. Second, our literature search may have missed relevant publications due to the nature of a scoping review. Finally, eligible studies may include biases due to study design and quality, definitions, and data sources.

### 4.2 Conclusion

Our findings suggest several implications for consideration and prioritization for appropriate therapeutic management of patients with ARIs who may be at high risk of complications. These include making consistent use of evidence-based protocols for testing, treatment, and follow-up; providing appropriate assessment and treatment for patients with risk factors for more severe disease; and ensuring evidence-based appropriate use of antibiotics and antivirals.

## Data Availability

The original contributions presented in this study are included in this article/Supplementary material, further inquiries can be directed to the corresponding author.
